# Impact of a senior research thesis on students' perceptions of scientific inquiry in distinct student populations

**DOI:** 10.1002/2211-5463.70145

**Published:** 2025-10-25

**Authors:** Celeste Suart, Haley L. Zubyk, Michelle Ogrodnik, Caitlin E. Mullarkey, Felicia Vulcu

**Affiliations:** ^1^ Department of Biochemistry and Biomedical Sciences McMaster University Hamilton Canada; ^2^ National Ataxia Foundation Minneapolis MN USA; ^3^ Department of Cell and Systems Biology, Human Biology Program University of Toronto Canada; ^4^ Department of Kinesiology and Health Sciences University of Waterloo Canada

**Keywords:** career goals, failure, research skills, resilience, scientific identity, undergraduate research experience

## Abstract

Senior research thesis courses are a hallmark feature of many undergraduate science programs, with several documented benefits, including the development of research skills and scientific identity alongside career exploration. In this study, we investigated how the senior research thesis experience is perceived by distinct student populations. We surveyed undergraduate students from two programs at a mid‐sized, research‐intensive university: Biochemistry, a basic science‐focused program, and Biomedical Discovery and Commercialization, a hybrid program combining science and business. Both groups were enrolled in identical fourth‐year laboratory‐based thesis courses. Our analysis measured the impact of the thesis experience on students' scientific inquiry skills and beliefs; furthermore, we examined how these changes influenced their professional socialization as researchers and their postdegree career goals. Our findings suggest that completing a senior research thesis increased students' perception of their research‐related skills, regardless of program enrollment. While there were fewer significant changes regarding student epistemological beliefs around scientific research, qualitative and quantitative measures support the idea that students have developed a more positive perception of failure and resilience within research. Additionally, while students within the science‐business hybrid program experienced no significant changes in career goals, completion of a senior research thesis had a significant impact on students within the science‐based program. Overall, our results demonstrate that laboratory‐based thesis courses can have a notable effect on developing student research skills, beliefs about scientific research, and career goals, and that these effects vary based on the student population.

## Graphical abstract

This study addressed how a senior research thesis is perceived by undergraduate students. It assessed students' perception of research skills, epistemological beliefs, and career goals in Biochemistry (science) and BDC (science‐business) students. Completing a thesis improved confidence in research skills, resilience, scientific identity, closed gender‐based confidence gaps, and shifted Biochemistry students toward research careers, highlighting the value of undergraduate research.

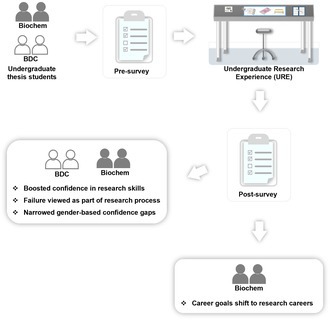

**Abbreviations**
BBS: Biochemistry and Biomedical Sciences
BDC: Biomedical Discovery and Commercialization
SAAB: Survey of Student Self‐Rated Abilities, Attitudes, and Beliefs
STEM: science, technology, engineering, mathematics
URE: undergraduate research experience

Undergraduate research experiences (UREs), such as laboratory project‐based senior research thesis courses, have become a defining feature of many undergraduate science degrees [1, 2]. Participating in research has consistently demonstrated multiple benefits for students, including the development of discipline‐specific research skills, improved critical thinking, enhanced problem‐solving abilities, as well as serving as a gateway to graduate research education [3, 4, 5]. URE participants have an increased likelihood of considering STEM graduate studies, research‐focused careers, and other STEM‐focused careers [6, 7, 8]. Furthermore, studies have shown that participation in undergraduate research significantly improves students' chances of acceptance into graduate research programs [9, 10]. This effect is particularly pronounced for students who engage in research for extended periods: longitudinal data indicate that those who participated in undergraduate research for at least two academic semesters were more likely to be accepted into STEM graduate training programs and to pursue STEM careers up to 6 years after completing their undergraduate education [11]. However, these benefits are not seen in students who leave their research experience earlier or view their completed research experience negatively [9, 12, 13]. Recently, there has been a shift in the literature to critically examine the educational components of an undergraduate research thesis, as well as how student demographic factors influence outcomes following research experiences [4, 14].

In line with this shift, there is growing attention to how UREs may help address long‐standing inequities in STEM education. Due to pervasive gender and racial disparities in students who enroll in science, technology, engineering, and mathematics (STEM) programs and complete STEM degrees, researchers have explored different educational interventions to increase the retention of underrepresented minorities within STEM programs, including UREs [15, 16, 17]. The completion of an URE has been shown to significantly influence women's decision to enter STEM graduate programs and secure employment in STEM fields [17, 18]. Similar increases have been identified in other marginalized groups, such as students of color, disabled students, and students from low socioeconomic backgrounds [16, 19, 20]. Many of these benefits have been attributed to forming a scientific identity during these experiences.

Forming a scientific identity involves integrating conceptual scientific knowledge, hands‐on research skills, and epistemological beliefs about scientific inquiry, which enable students to envision themselves as researchers [21, 22]. This identity is strengthened through acquiring new knowledge and skills, engaging in scientific discourse with peers and researchers, and receiving feedback or recognition for their efforts from senior scientists—all common elements of UREs [8]. Developing a scientific identity is a complex process requiring students to reconcile their evolving scientific identity with their personal identity. It also involves navigating challenging transitions, such as embracing frequent failures and honing troubleshooting skills [8, 12, 23]. High‐quality mentorship from senior researchers plays a crucial role in helping students manage these transformative and often demanding experiences [24, 25].

At McMaster University, the Department of Biochemistry and Biomedical Sciences (BBS) offers students in its undergraduate programs the opportunity to complete a fourth‐year research‐based thesis project. These thesis courses span two main program streams: the Biochemistry programs and the Biomedical Discovery and Commercialization (BDC) program. The Biochemistry program is a Level II entry program and focuses extensively on developing biochemistry knowledge and research skills. All Biochemistry students complete a mandatory 8‐month second‐year laboratory course (BIOCHEM 2L06), which introduces them to inquiry‐based learning and core biochemistry research techniques, with the option to take an additional third‐year laboratory course (BIOCHEM 3LA3), which focuses on advanced biochemistry laboratory techniques. In contrast, the BDC program is a Level III entry program (that accepts students from biochemistry and other academic disciplines) and integrates biochemistry training with business intelligence. BDC students complete a mandatory 8‐month third‐year laboratory course (BIOMEDDC 3C09), which emphasizes techniques used in drug discovery. Like the previous two laboratory courses, BIOMEDDC 3C09 focuses students on both fundamental and advanced biochemistry laboratory skills with a strong emphasis on how these techniques are used in research projects. Some BDC students have little to no biomedical laboratory experience prior to this course. However, other students transfer from the Biochemistry program into BDC after their second year, giving them the opportunity to complete both BIOCHEM 2L06 and BIOMEDDC 3C09, thereby gaining substantial course‐based research experience before starting their fourth‐year thesis.

In both streams, the thesis courses are 8‐month capstone experiences designed to accommodate the differing course‐based needs of their students. In the Biochemistry program, students have the option of pursuing this capstone experience under two course options BIOCHEM 4F09 and 4T15. These courses only differ in credit value, which corresponds to the expected number of hours students spend in the laboratory each week. On the other hand, BIOMEDDC 4A15 is offered to students in the BDC program. Despite the different codes, all three thesis courses have identical learning objectives, which emphasize advanced research skill development, transferable skills, and self‐directed learning. As such, to ensure consistency, the courses are administered under a unified course syllabus.

In this study, we investigated the impact of participating in a senior biomedical research thesis on students' abilities, attitudes, and beliefs about scientific research. The study focused on these three jointly administered thesis courses (BIOCHEM 4F09, BIOCHEM 4T15, and BIOMEDDC 4A15) during the 2022–2023 academic year, comparing students from two distinct programs: Biochemistry (science‐focused) and BDC (a science‐business hybrid). Our findings revealed that completing a senior research thesis significantly enhanced students' research skills; however, changes in beliefs and attitudes about scientific inquiry were less pronounced across both student populations. Notably, the thesis experience eliminated gender‐based differences in perceived abilities: women, who rated themselves lower than men in various skills at the outset, reported comparable abilities to men by the end of the course. Interestingly, participation in a senior thesis appeared to influence career goals differently between programs, with Biochemistry students reporting greater shifts in their career aspirations, while the goals of BDC students remained largely unchanged. These results add to the growing body of literature supporting the role of a senior research thesis in the development of research skills and positively impacting the formation of a scientific identity, particularly for equity‐deserving groups. Our results additionally suggest that UREs can influence career goals unevenly across disciplines, which highlights the need to tailor research experiences to better support students' professional development.

## Methods

This study and consent protocol was approved by the Hamilton Integrated Research Ethics Board (HiREB) under project number 15082 on 6 September 2022.

### Survey questions

The Pre‐ and Post‐Thesis Survey instruments comprised four sections: survey ID generation, demographic questions, the Survey of Student Self‐Rated Abilities, Attitudes, and Beliefs, and open‐ended questions. Quantitative questions within the survey were mandatory to complete, while qualitative questions were optional.

In both surveys, respondents were guided in the survey ID generation section to develop a self‐generated identification code to link their Pre‐ and Post‐Thesis Survey responses [26]. This was a series of four questions to generate a 12‐digit alphabetical code.

The demographics section of the Pre‐Thesis Survey asked multiple‐choice or open‐ended questions on respondent gender, thesis course (BIOCHEM 4F09, BIOCHEM 4T15, or BIOMEDDC 4A15), past laboratory experience, and current career goals. A shortened demographics section was included in the Post‐Thesis Survey, including questions on gender, thesis course, and change in career goals.

The third section of the Pre‐ and Post‐Thesis Survey was the Survey of Student Self‐Rated Abilities, Attitudes, and Beliefs (SAAB), a previously validated scale for assessing student attitudes, abilities, and beliefs about the scientific process developed by Hoskins and colleagues [27, 28]. This Likert‐style instrument contains statements relating to their self‐efficacy regarding research activities and epistemological beliefs relating to the nature of scientific research. Measures of self‐efficacy include decoding literature, interpreting data, active reading, visualization, and understanding research in context [28]. Measures relating to epistemological beliefs include the certainty of knowledge, if ability is innate, if science is creative, what scientists are like as people, the motivations of scientists, if the outcomes of experiments should be predictable, and if scientific inquiry should be collaborative [28].

To provide additional context to demographic and SAAB responses, we included open‐ended questions for students to provide narrative responses. For example, in the Pre‐Thesis Survey, we asked the respondents, ‘What are three things they hope to gain from their senior thesis?’ Additionally, in the Post‐Thesis Survey, we asked, ‘What are three things they learned about being a biomedical researcher’. Finally, we queried respondents about how their thesis experience impacted their career, with options for respondents who indicated their thesis experience solidified their career goals and those who changed their career goals. Pre‐ and Post‐Thesis Survey design, questions, and question response formats are included in Tables S1 and S2.

### Survey administration

McMaster University is a research‐intensive public university in Hamilton, Ontario, Canada. All students enrolled in BIOCHEM 4F09: Senior Thesis, BIOCHEM 4T15: Senior Thesis, and BIOMEDDC 4A15: Senior Research Thesis in the 2022–2023 academic year were invited to participate in this study. BIOCHEM 4F09 and BIOCHEM 4T15 are senior undergraduate research courses available to students in the Biochemistry programs. At the same time, BIOMEDDC 4A15 serves students in the BDC program, which combines biochemistry training with business acumen. These fourth‐year, research‐based thesis courses are jointly administered, with identical student deliverables and assessment methods across all three courses. The thesis courses vary in credit value and required in‐lab time, providing flexibility for students to tailor their fourth‐year academic experience. Spanning two academic terms (8 months, fall and winter), the courses focus on a literature review (20%), laboratory performance assessments throughout the course (40%), a written thesis (20%), and a final oral presentation delivered to a panel of researchers (20%). Participation in this study was open to all enrolled students, with no inclusion or exclusion criteria based on age, citizenship, ethnicity, disability, gender, or race.

Participants were asked to complete two anonymized online surveys as part of the study, one in September 2022 before beginning their senior research thesis (Pre‐Thesis Survey), and one in March 2023 at the end of their thesis experience (Post‐Thesis Survey). Each survey had a two‐week advertisement period, 12 September 2022 to 23 September 2022 and 13 March 2023 to 24 March 2023. Information about both surveys was shared with all thesis students (*n* = 118) via email through their program administrator and posted to the unified course learning management system for BIOCHEM 4F09 (*n* = 5), BIOCHEM 4T15 (*n* = 58), and BIOMEDDC 4A15 (*n* = 55). Surveys were administered through LimeSurvey, taking approximately 15–20 min to complete. After completing each survey, students were given the option to be entered into a draw for one of ten $50 UberEats gift cards.

The Pre‐Thesis Survey had a 50% response rate (*N* = 59), while the Post‐Thesis Survey response rate was 36% (*N* = 42). Thirty‐three respondents completed both the Pre‐ and Post‐Thesis Surveys. Demographic characteristics across the Pre‐ and Post‐Thesis Surveys were similar (Table 1). Slightly more women than men responded to the survey, and over half of the respondents were enrolled in biochemistry thesis courses (BIOCHEM 4F09 or 4T15) (Table 1). These slight skews in numbers reflect the trends within the total student populations in these three thesis courses. It is important to note that in the demographic portion of the survey, participants were asked, ‘How would you describe your gender?’ The response format was an open‐ended free‐text box, and the question was mandatory, though students could opt out by entering ‘N/A’ (Tables S1 and S2, Demographic Questions). Although students were asked about gender, all respondents identified themselves using sex‐based terms, specifically ‘male’ and ‘female’. For the purposes of this study, these sex‐based responses were interpreted in a gendered context, with ‘male’ equating to ‘man’ and ‘female’ equating to ‘woman’. There were no significant differences in the genders of students registered in each thesis course, and no significant differences in the genders of respondents to this survey when stratified by thesis course.

**Table 1:** Survey respondent characteristics.

Characteristics	Pre‐thesis survey, *N* (%)	Post‐thesis survey, *N* (%)
Gender		
Male	26 (44)	18 (43)
Female	33 (56)	24 (57)
Thesis course		
BIOCHEM 4F09	3 (5)	2 (5)
BIOCHEM 4T15	32 (54)	24 (57)
BIOMEDDC 4A15	24 (41)	16 (38)

To better understand the impact of past laboratory experiences on students entering senior research thesis courses, we asked respondents about previously completed laboratory‐focused courses and any prior work, volunteer, or educational experience they had in research laboratories (Table 2).

**Table 2:** Past laboratory experiences of respondents before their senior research thesis.

Prior experience	Pre‐thesis survey, *N* (%)
Past laboratory courses	
BIOCHEM 2L06	52 (88)
BIOCHEM 3LA3	12 (20)
BIOMEDDC 3C09	24 (41)
Other senior‐level laboratory courses	0 (0)
Past research experience	
Worked or volunteered in their current thesis laboratory	27 (46)
Worked or volunteered in another laboratory	13 (22)
Completed a third‐year research thesis	15 (25)
Other laboratory experience	0 (0)

For prior laboratory courses, 88% of respondents had taken the mandatory second‐year introductory Biochemistry laboratory course (BIOCHEM 2L06), 20% had taken an elective third‐year advanced Biochemistry laboratory course (BIOCHEM 3LA3), and all BIOMEDDC 4A15 thesis students (41%) had taken the mandatory third‐year introductory BDC course (BIOMEDDC 3C09) (Table 2). As BDC is a third‐year entry program, with some students transferring from the biochemistry program, 29% of respondents had completed both BIOCHEM 2L06 and BIOMEDDC 3C09.

We then asked respondents about past experiences in research laboratories lasting 8 weeks or longer. 31% of respondents had no laboratory research experience prior to completing their senior research thesis, with the remaining two‐thirds completing third‐year research courses or having work or volunteer experience in research laboratories (Table 2).

### Data analysis

Statistical analyses were performed in graphpad prism 9 (GraphPad Software, Inc., San Diego, CA, USA) and Microsoft Excel (Microsoft Corporation, Redmond, WA, USA). Descriptive statistics were generated for demographic and career goal questions. Analysis of the SAAB instrument data followed protocols described by Hoskins and colleagues [27, 28]. The SAAB subscales consist of items measured on one or more 5‐point Likert scales (ranging from *strongly disagree* to *strongly agree*). Because Hoskins *et al*. [28] applied principal component analysis to refine the SAAB tool to the most sensitive items, the total possible scores vary across subscales. However, for all SAAB subscales, a higher score indicates responses more aligned with those of biomedical scientists, whereas lower scores reflect patterns more typical of students with little prior research experience [27, 28].

The mean and standard deviation for each SAAB subscale score were calculated and stratified by demographic data. To analyze SAAB subscale scores, normality was assessed using the Shapiro–Wilk test. For nonparametric data, the Mann–Whitney *U* test was applied for total population samples, or Multiple Mann–Whitney analysis for data stratified by demographic factors. For parametric data, paired Student's *t*‐tests were used for total population samples, or 2‐way ANOVA for stratified data [27, 29]. Cohen's effect size was calculated to determine whether statistically significant changes corresponded to meaningful educational outcomes [30]. To evaluate whether thesis participation influenced students' career goals, nominal data from related samples were analyzed using the McNemar change test [31]. For all graphs, error bars represent the minimum and maximum values, interquartile range, and median.

We employed a qualitative content analysis approach to assess qualitative data, with a hybrid inductive and deductive approach [32]. Two of the authors separately developed codes using inductive content analysis. Following the initial analysis, all codes were reviewed for agreement, with discrepancies resolved through discussion until a consensus was reached. This master coding list was given to a third independent author to verify whether an individual who has not previously worked with the data could identify the same proportion of codes. Only codes with frequencies greater than or equal to 15% of responses were included for further analysis.

Overall, we employed a convergent mixed‐methods analysis of the qualitative and quantitative data [33]. Through this analytical approach, we conducted a side‐by‐side comparison of quantitative and qualitative results. We identified convergent and divergent findings of data examining the same phenomenon through different means.

## Results

### Prethesis baseline characteristics

**Fig. 1:**
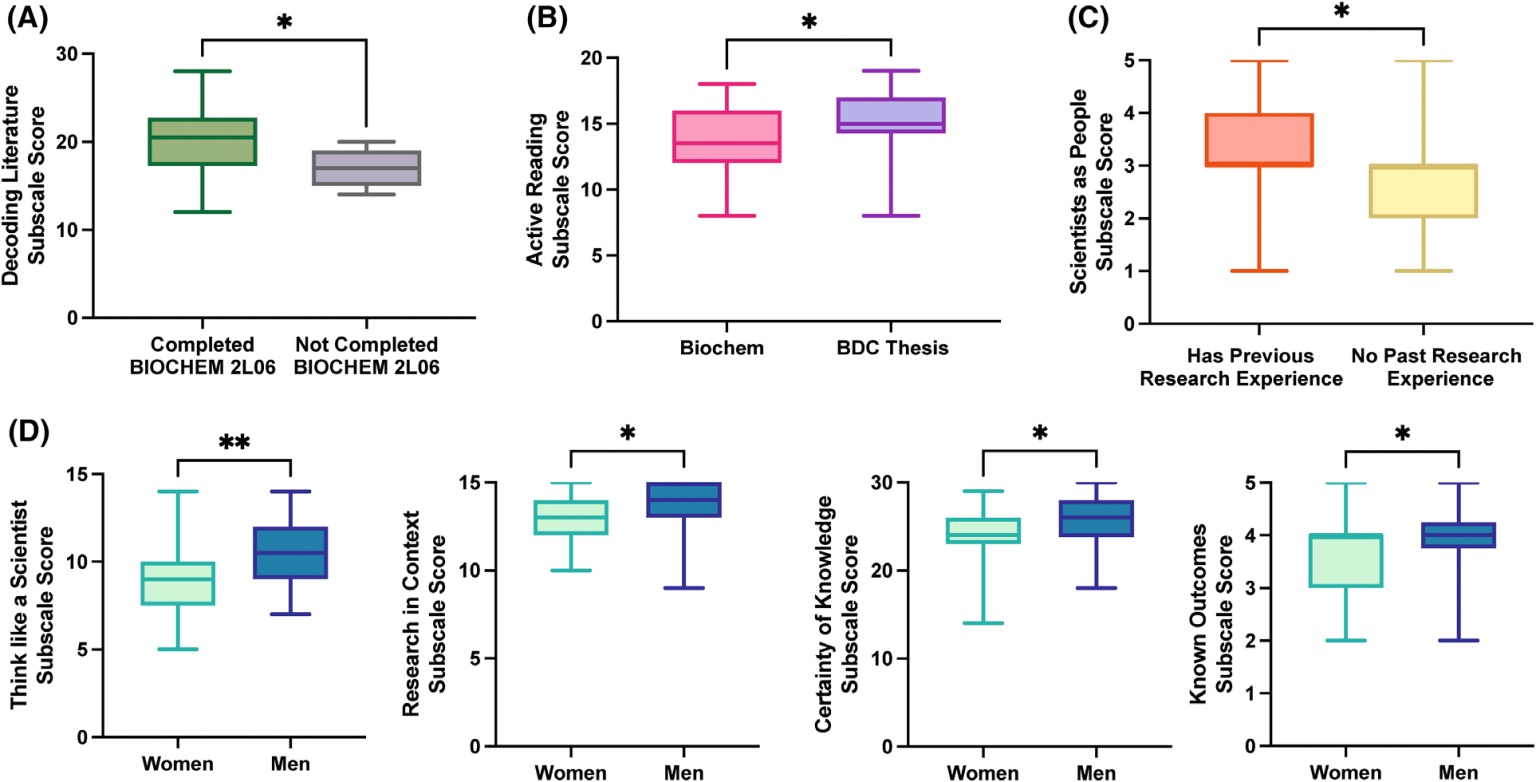
Subpopulation differences between students entering senior research thesis courses. *N* = 59. (A) Subpopulation differences when stratified by prior laboratory course experience. (B) Subpopulation differences when stratified by current thesis course. (C) Subpopulation differences when stratified by prior research experience. (D) Subpopulation differences when stratified by gender. Mann–Whitney, *P < 0.05, **P < 0.01.

We first explored whether baseline differences in research skills and epistemological beliefs about sciences existed between cohorts. When stratifying students by prior laboratory course experiences, we found respondents who had completed BIOCHEM 2L06, a mandatory second‐year introductory biochemistry laboratory course, were significantly more comfortable decoding scientific literature (*P* = 0.0392, Fig. 1A). This population represents students in both the biochemistry stream and BDC students who had completed the biochemistry curriculum before transferring to the BDC program. Respondents from the BDC thesis course reported greater confidence in assessing graphs and research methodology (*P* = 0.0184, Fig. 1B).

These results were not wholly unexpected given the learning objectives and structure of BIOCHEM 2L06 and BIOMEDDC 3C09. Both courses were designed and taught by the same instructor, with many unified learning objectives that emphasize not only biochemistry technical skills but also transferable skills such as experimental design, teamwork, critical thinking, resilience, collaboration, and scientific communication.

BIOCHEM 2L06 was designed to guide second‐year biochemistry students through an established research project focused on characterizing a protein of interest. The instructor guides students through molecular cloning and protein purification techniques while emphasizing each technique, from protocol design to data analysis. Similarly, BIOMEDDC 3C09 focuses third‐year students on core and advanced biochemistry laboratory skills and provides a firm foundation in building a framework for tackling experimental design. While the BIOMEDDC 3C09 course content delves far deeper into technical concepts, both courses aim to demystify the research process and help students feel confident and competent in a laboratory setting. The learning objectives shared by both these courses likely contributed to the observed findings, particularly in preparing students for their fourth‐year thesis projects.

No further population differences were identified based on past or present course enrollment. Perhaps unsurprisingly, respondents with no prior research experience indicated they were less familiar with what scientists were like as people (*P* = 0.0235, Fig. 1C).

Gender was tested for all aspects of the survey. Of these, significant differences were only observed for the subscales of Thinking like a scientist (*P* = 0.0057), Research in Context (*P* = 0.0317), Certainty of knowledge (*P* = 0.0377), and Known Outcomes (*P* = 0.0419) (Fig. 1D). These findings are consistent with the previous literature documenting that women in STEM fields have lower self‐evaluations of their abilities, while men tend to overestimate their abilities [34, 35, 36].

Next, we queried respondents about what they hoped to gain from their senior research thesis experience (Table 3). The most common responses focused on gaining a better understanding of the research process and how research takes place (42%), followed by hoping to develop better critical thinking skills (37%). Other respondents identified developing technical research‐related skills (26%), gaining insight into laboratory‐based graduate school or future career options (24%), improving experimental design skills (24%), and networking with potential mentors (21%) as key hopes for their thesis experience. A minority of respondents indicated that they were looking forward to having laboratory research experiences on their resumes for career building (15%) or looking for specific knowledge building in a particular field of study (15%).

**Table 3:** What respondents hoped to gain from their senior research thesis experience at the start of the year (*N* = 38).

Theme	*N* (%)	Representative quotations
What research is like	16 (42)	‘I hope to have a better grasp of how researchers come up with research topics and methods’ ‘Insight into how everything works in research labs’ ‘Experience drafting a manuscript for publication’
Critical thinking skills	14 (37)	‘Become better at critical thinking (asking more insightful questions about research, challenging what's already known etc.)’ ‘Learn how to critically analyze scientific literature’
Research skills	10 (26)	‘Strong technical skills in a variety of biochemical lab techniques’ ‘Better science communication skills in a written format’
Insight into graduate school or future career options	9 (24)	‘I am planning on not letting the thesis “make” me someone who likes research, but shows me if I would find it interesting as a career’ ‘Getting a glimpse of what graduate school is like’
Experimental design	9 (24)	‘Improve my experimental design skills’ ‘Better creativity in creating experiments’
Mentorship and networking	8 (21)	‘I hope to gain a strong mentor(s) that I can contact years from now’ ‘A strong letter of recommendation for graduate school’
Experience in a research laboratory	5 (15)	‘I want to have wet lab experience because that may aid me going forwards’
In‐depth knowledge of a research topic or field of study	5 (15)	‘Learning a lot about my field of interest’ ‘Gain a comprehensive understanding in the field of *[Identifiable Subject Area]* and related disciplines’

### Comparison of pre‐ and post‐thesis survey results

**Fig. 2:**
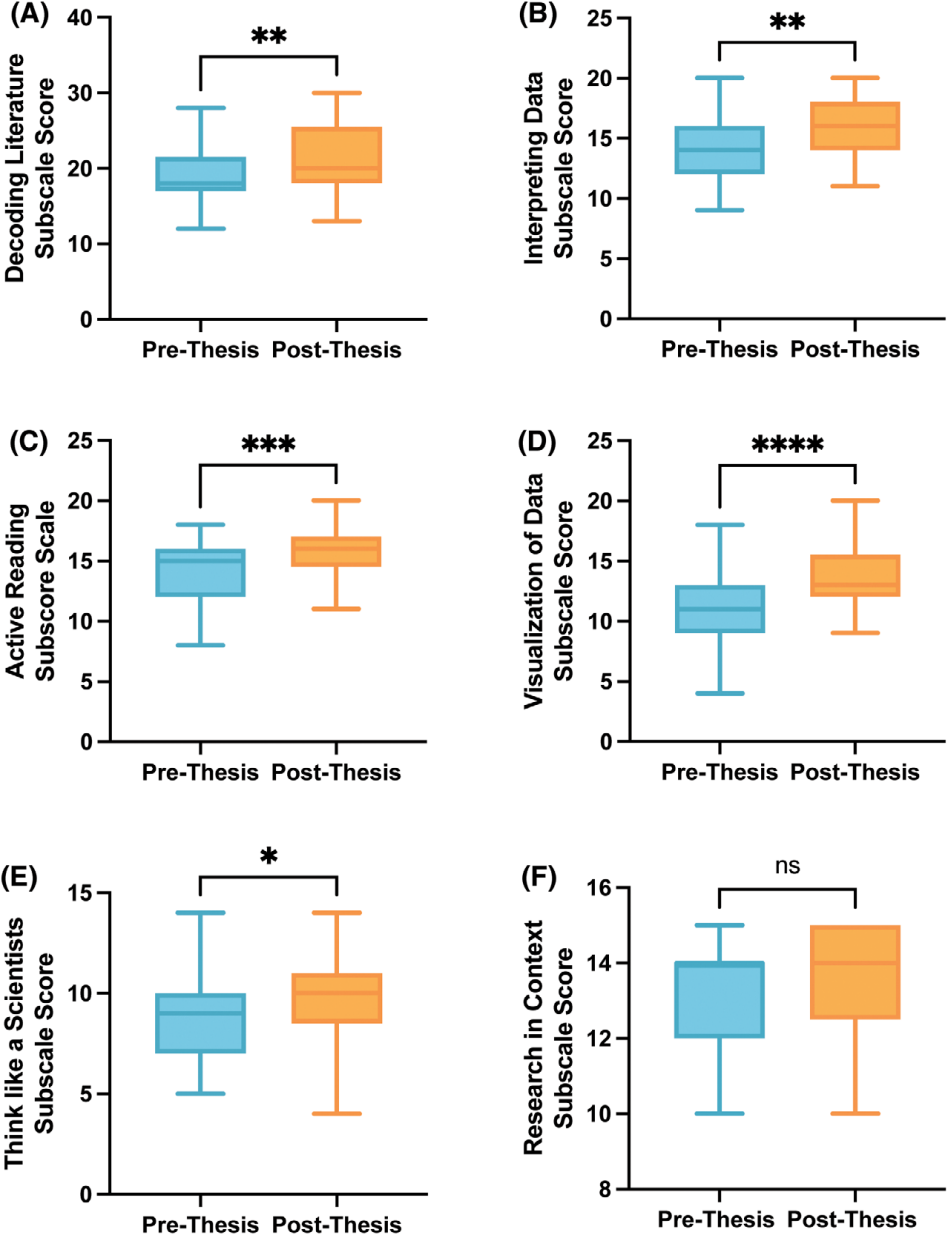
Change in research skill self‐efficacy ratings following research thesis. *N* = 33. (A) Decoding literature subscale. (B) Interpreting data subscale. (C) Active reading subscale. (D) Visualization of data subscale. (E) Thinking like a scientist subscale. (F) Research in context subscale. Paired Student's *t*‐test, *P < 0.05, ****P < 0.0001.

**Fig. 3:**
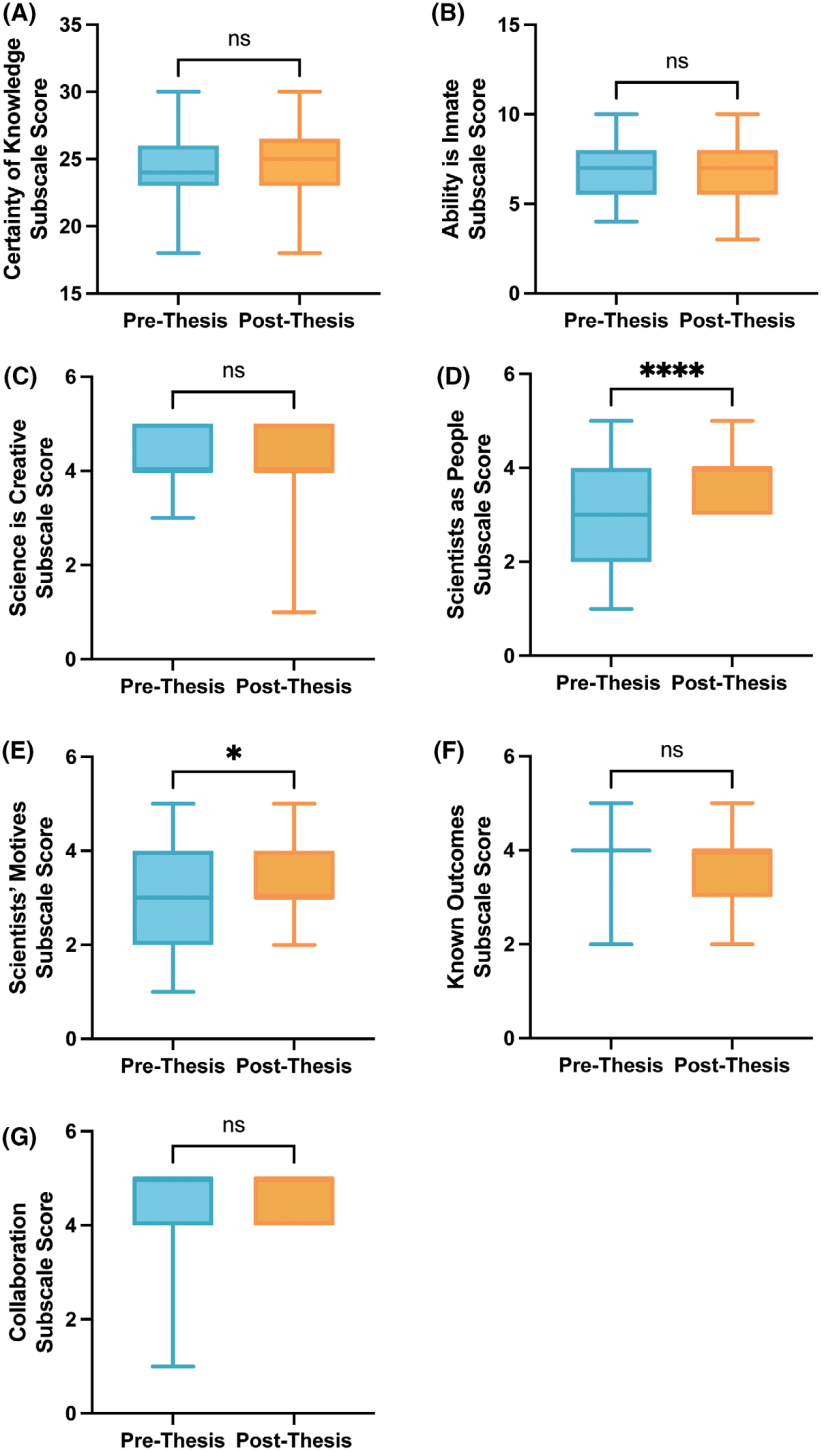
Change in epistemological beliefs ratings following research thesis. Pre‐thesis *N* = 33. (A) Certainty of knowledge subscale. (B) Ability is innate subscale. (C) Science is creative subscale. (D) Scientists as people subscale. (E) Scientists' motives subscale. (F) Known outcomes subscale. (G) Collaboration subscale. Paired Student's *t*‐test, *P < 0.05, **P < 0.01, ***P < 0.001, ****P < 0.0001.

Following the completion of the research thesis courses, we compared students' prethesis SAAB subscale scores with post‐thesis subscale scores (Figs 2 and 3). Respondents had significant increases in subscales relating to research skill self‐efficacy, including decoding scientific literature (*P* = 0.0011, Cohen's *d* = 0.46), interpreting data (*P* = 0.0020, Cohen's *d* = 0.53), active reading (*P* = 0.0005, Cohen's *d* = 0.67), visualizing data (*P* < 0.0001, Cohen's *d* = 0.88), and thinking like a scientist (*P* = 0.0405, Cohen's *d* = 0.33) (Fig. 2). However, there were no significant improvements to Research in Context subscale scores (*P* = 0.1644), which relate to understanding why experiments have controls and why animal models are used in research (Fig. 2).

Fewer changes were observed in students' epistemological beliefs about science (Fig. 3). Although respondent scores indicated an increase in their understanding of scientists as people (Fig. 3D, *P* < 0.0001, Cohen's *d* = 1.04) and scientists' motivations (Fig. 3E, *P* = 0.0465, Cohen's *d* = 0.41), there were no other significant differences between prethesis and post‐thesis epistemological belief subscale scores for the total student population.

**Fig. 4:**
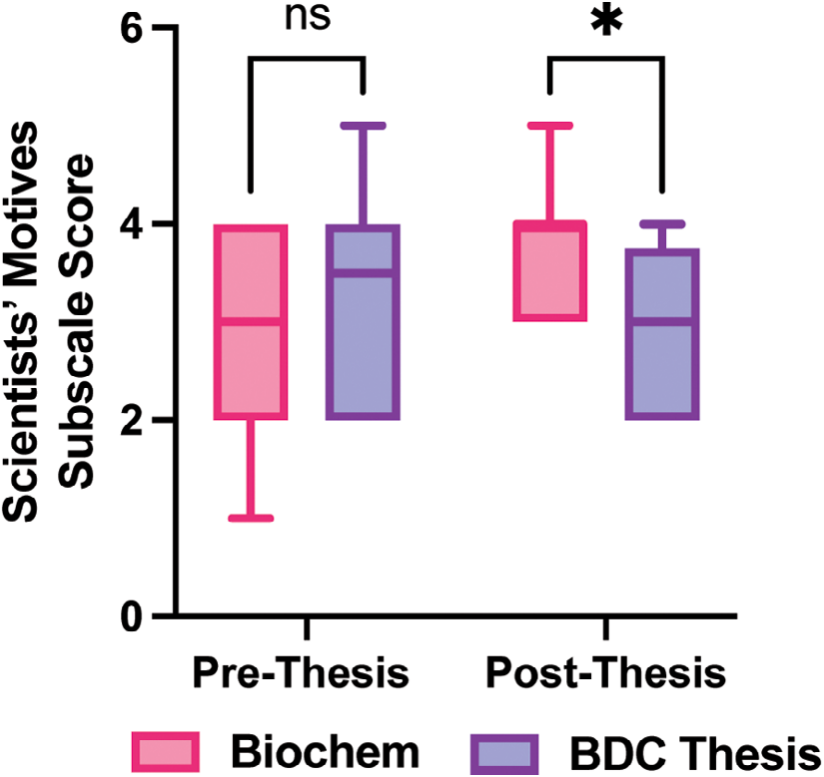
Subpopulation differences between students following the senior research thesis. *N* = 48. Scientists' motivations subscale. Multiple Mann–Whitney, *P = 0.0134.

Next, we examined whether subpopulation differences observed at the beginning of the research thesis experience were present following the completion of the thesis course. With one exception, there were no significant differences between subpopulation scores following the research thesis experience, including when stratified by current thesis course, gender, previous research experience, or previous course experience. However, a new subpopulation difference emerged post‐thesis when stratifying subscale scores by thesis course; students registered in the biochemistry thesis courses reported a significantly higher understanding of scientists' motives than those in the BDC thesis course (Fig. 4, *P* = 0.0134).

Mirroring our open‐ended question prior to the thesis experience, we asked participants what they learned from completing their research thesis experience (Table 4). Post‐thesis responses had greater consolidation of main themes, with 84% of respondents indicating they developed a better understanding of the research process and what being a researcher is like, 53% recounting moments of failure and resilience during research, and 47% sharing that they learned about what makes a good biomedical researcher. Less frequently represented themes included the development of critical thinking and research skills (32%), learning the importance of collaboration and teamwork (32%), and developing experimental design skills (16%).

**Table 4:** What respondents learned about being a biomedical researcher during their senior research thesis experience (*N* = 19).

Theme	*N* (%)	Representative quotations
What research is like	16 (84)	‘Optimization of experimental protocols takes much more time than I thought before’ ‘I've learned how hard and time‐consuming it is to develop and run an experiment’ ‘The work is schedule‐less, especially when working with cell cultures (i.e. the cells determine your schedule), and the hours in a working day vary quite a lot, and are somewhat unpredictable’
Failure and resilience	10 (53)	‘I've learned that failure is an innate and important part of science’ ‘The polished stories in published papers are the result of much, often hidden, failure and hard work’
What makes a good researcher	9 (47)	‘Being a biomedical researcher requires a deep passion for the field for it to be enjoyable in the long term’ ‘It takes a lot of patience as more things go wrong than right’ ‘Scientists get very excited about their research!’
Critical thinking and research skills	6 (32)	‘I have learned the importance of critically analyzing and interpreting data even from published papers’ ‘Important basic (and more complicated) lab skills’
Importance of collaboration and teamwork	6 (32)	‘Laboratories are extremely collaborative and interdisciplinary environments’ ‘For things to go well, it is better to work with other people where you can all contribute with your strengths’
Experimental design	3 (16)	‘Controls are important, getting a good control is more satisfying than actually doing the experiment, because when a control is successful it validates your method that you developed’ ‘Repetition of experiments is your friend’

### Thesis participation impact on career trajectory

**Fig. 5:**
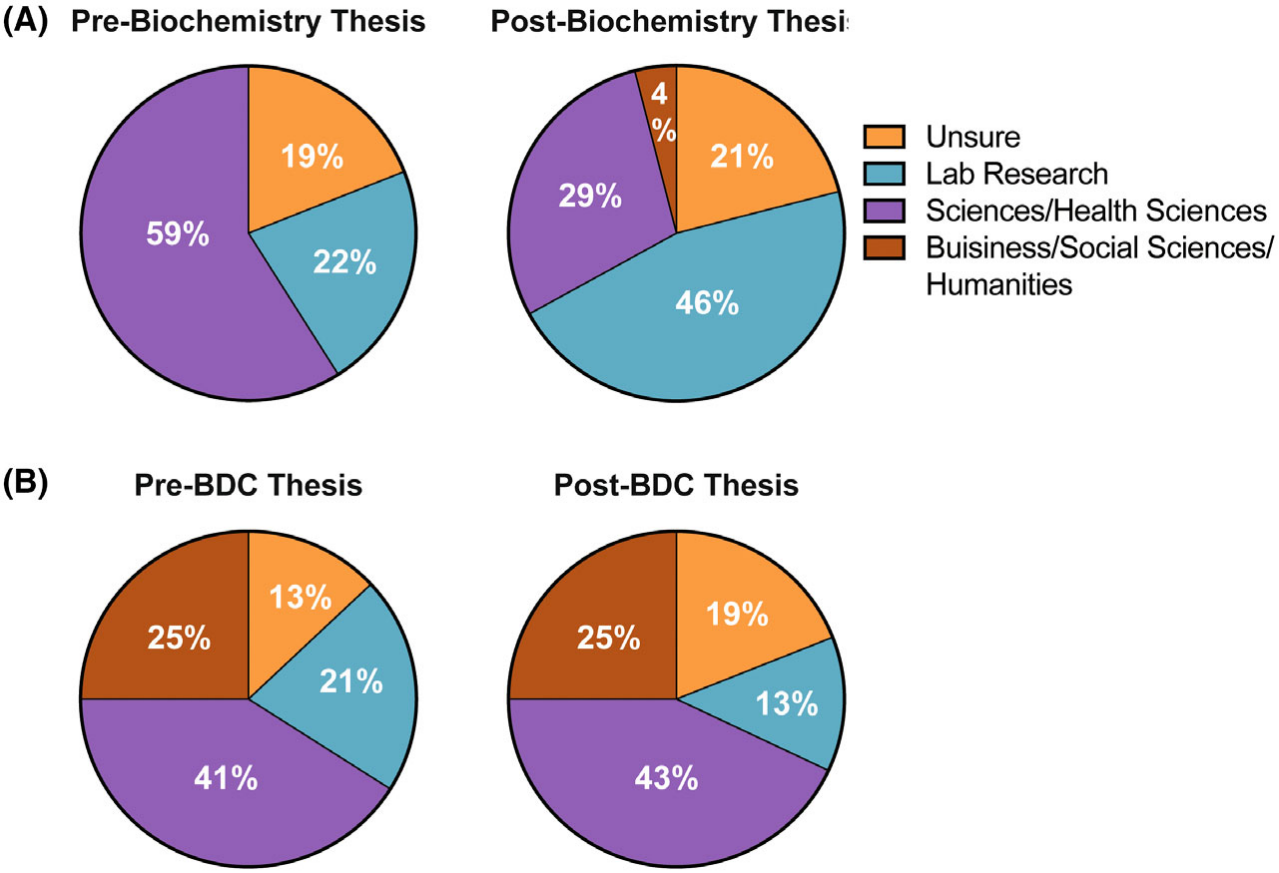
Respondent career goals pre‐ and post‐thesis experience. (A) Biochemistry student respondents, *N* = 26–35. (B) Biomedical Discovery and Commercialization student respondents, *N* = 16–24.

As UREs are known to impact participant career aspirations [6, 37], we were interested in how participating in a senior research thesis would impact the career goals of our respondent cohort. Prior to the thesis experience, significantly more BDC students were considering careers in business, social sciences, or humanities than biochemistry students (Fisher's exact test, *P* = 0.0049, Fig. 5). This difference between populations is not surprising given that the BDC program is interdisciplinary and merges biochemistry and innovation and commercialization skills. However, for both subgroups, the majority of respondents were interested in nonlaboratory careers in science or health science (Fig. 5).

These trends in career goals changed following the thesis experiences. Although the proportions of respondents' career goals remained similar for BDC respondents (McNemar's Test, *P* = 0.317), significantly more biochemistry respondents became interested in laboratory‐based careers (McNemar's Test, *P* = 0.0455). This suggests that completing a senior research thesis affected biochemistry students' career goals (Fig. 5). Nonetheless, this impact only seems to be present for respondents who had a known career goal prior to completing their senior thesis, as there was no significant change in the proportion of respondents who were unsure of their career goals (McNemar's Test, *P* = 0.7055). Women were also more likely than men to keep the same career goal or uncertainty about their career goal from before completing their thesis experience, regardless of what the career goal was (McNemar's Test, *P* = 0.0339).

We asked respondents whose career aspirations were either modified (Table 5) or confirmed (Table 6) by their senior research thesis to elaborate on how this experience impacted their career goals. Most respondents who said their thesis modified their career trajectory were those who changed from initially aspiring to laboratory‐based careers to nonlaboratory‐based careers. They described similar sentiments, as one respondent put it, ‘I realized that research might not be for me’. Others specifically mentioned how they realized that laboratory‐based careers would not be a good fit for them after gaining first‐hand experience (Table 5). Feedback from respondents who felt their thesis experience had confirmed their career choice was more diverse—55% discussed how this experience confirmed they enjoy the nature of laboratory work and 27% mentioned how completing their thesis reinforced their preference for a nonlaboratory‐focused career path (Table 6). Similar to those who modified their career goals, 18% of respondents indicated their thesis experience confirmed that they dislike laboratory research (Table 6).

**Table 5:** Respondents whose career goals were modified by their senior research thesis experience (*N* = 9).

Theme	*N* (%)	Representative quotations
Discovering research is not for me	6 (67)	‘My thesis has made me realize that while research is interesting, it is not necessarily the career that I would consider as my first option’ ‘More hesitant about going into research’
Other	3 (33)	‘I developed a new appreciation for research’ ‘I thought I would go into academia, but instead I realized I would rather go into industry’

**Table 6:** Respondents whose career goals were confirmed by their senior research thesis experience (*N* = 11).

Theme	*N* (%)	Representative quotations
Confirming research is for me	6 (55)	‘Although I was leaning towards research, I was uncertain if it was the right pathway for me. Doing a thesis and the accompanying excitement solidified this career goal for me’ ‘It confirmed that I love research and want to do grad school’
Confirming another field is for me	3 (27)	‘It has solidified what I am interested in (the “commercialization side”, including pharma sales and consulting for pharma companies)’ ‘Since I am planning to enter the field of *[Identifiable Medical Subspecialty]*, this research project has definitely affirmed my decision to enter this field’
Confirming research is not for me	2 (18)	‘I definitely do not want to go into research’

## Discussion

In this mixed‐methods study, we examined the impact of a senior research thesis on students' development of research skills, epistemological beliefs about scientific research, and career goals. This was achieved through the use of the SAAB survey tool, quantitative demographic questions, and open‐ended qualitative questions. While these topics have been studied individually in the context of UREs, a unique strength of this investigation was to integrate all three in a single line of inquiry with a large cohort (*n* = 118 students enrolled in all programs, *n* = 33 students who responded to both surveys) of fourth‐year thesis students. Moreover, by surveying students enrolled in separate programs, our research offers novel insights into how distinct student populations are differentially impacted by an identical senior research thesis experience. Our data are consistent with previous findings on how students enter thesis experiences with diverse backgrounds, understanding of science, and familiarity with research [8, 11, 38]. Though there have been previous calls for examination into how UREs impact students with distinct interests, lived experiences, and preparation, there has been limited exploration of these factors [22]. Our findings demonstrate how subpopulation differences can be present within thesis cohorts due to previous instruction and program emphasis, past research experience, and student confidence. Identifying and understanding these differences in research skills and beliefs offers the opportunity for curricular design to meet students where they are, such as laboratory skill bootcamps or providing drop‐in workshops on reading scientific literature and data analysis. There is also the potential for scaffolded and targeted mentorship from thesis supervisors to help students develop their scientific identity and epistemological beliefs regarding research [24, 25].

Further, we captured a snapshot of how participation in a senior research thesis impacts research skills development and improves research self‐efficacy [22]. Our participants saw marked increases in multiple research skill subscales of the SAAB throughout their thesis, with several having medium‐to‐large effect sizes. Together, these data suggest that participants' thesis experience was effective in developing their research skills, or at least their self‐perception of efficacy at these research tasks. This trend is further reinforced through qualitative feedback from pre‐ and post‐thesis responses, where students mentioned the development of specific research skills, such as critical thinking and experimental design.

However, the qualitative and quantitative data diverge regarding changes in participants' epistemological beliefs. Overall, there were limited changes between pre‐ and post‐thesis subscale scores regarding attitudes and beliefs about scientific research. However, significant quantitative changes were recorded regarding the understanding of scientists as people and scientists' motivation. This aligns with the qualitative feedback of prethesis participants desiring to understand what research is like and post‐thesis participants' commentary on the nature of research, failure, and resilience in science, and what makes a good researcher. We should note that the manner in which we phrased our open‐ended questions and conducted our analysis was not exhaustive; we did not ask participants to share everything they hoped to gain in their thesis experience nor all they ended up learning. Rather, the themes shared in this paper reflect what skills and beliefs students judge as most important—which through their own words emphasize soft skills and understandings of research more frequently than hard skill development. Taken together, these findings suggest that while measurable shifts in epistemological beliefs were limited, students' reflections highlight meaningful growth in their perceptions of the research process and the human dimensions of scientific work. In particular, our data emphasize the value students place on personal insight, resilience, and the broader context of doing science.

Though not explicitly measured within the SAAB survey tool, over half of the qualitative comments regarding ‘what participants learned about being a researcher during their thesis’ discussed failure and resiliency. Failure is a critical part of the scientific process, yet many undergraduate science students have negative perceptions of failure due to stigma and negative connotations prevalent in academic and nonacademic environments [23, 39]. Moreover, there are currently limited opportunities for students to positively engage with failure within STEM undergraduate curricula outside of thesis experiences [23, 40]. As productive failure and resiliency are key components in the development of STEM professionals [41, 42], the shift in qualitative responses from participants to positive discussion of failure shows a noteworthy change in perceptions.

Other large cohort studies of UREs have shown an impact on participants' career exploration, STEM career selection, and pursuit of STEM graduate studies [8, 11, 22]. Though many of our participants were already considering research and related STEM careers prior to their thesis experience, we did see a significant shift in participants from the biochemistry thesis stream from professional STEM careers such as medicine or dentistry to laboratory research‐focused careers. We did not see this same shift in BDC thesis stream participants, as this cohort's career goals remained consistent pre‐ and post‐thesis experience. We propose that this difference may be attributed to the inherent structure and educational focus of each program. The biochemistry program allows for greater curricular flexibility and broader exploration of biochemical and biomedical career pathways throughout the undergraduate degree, which may leave more room for experiences like a senior thesis to influence career trajectories. In contrast, the BDC program is more structured with a predefined combination of biomedical science and applied business training. As such, students enter the BDC later in their academic careers (Level 3 as compared to Level 2 in Biochemistry) and may do so with clearer career goals and stronger pre‐existing alignment between their academic choices and professional aspirations. This could inherently reduce the likelihood of a major shift following their thesis experience. Another explanation for the career goal shift differences between thesis streams could also be the post‐thesis difference in understanding scientists' motivation. BDC respondents had similar levels of understanding pre‐ and post‐thesis, while biochemistry respondents reported significant increases in the scientists' motivations subscale post‐thesis experience. This deeper understanding of what motivates scientists may be what is encouraging participants to seek out similar careers in research. Nevertheless, this finding has implications for more specialist STEM programs as they design thesis experiences, as they could potentially include content on known specific careers of interest for students, rather than emphasizing exploration of options. For example, thesis courses in specialist STEM programs might integrate modules or guest lectures focused on industry‐specific roles, such as regulatory affairs in biotechnology, data science applications in genomics, or clinical trial coordination in pharmaceutical development. By aligning thesis experiences more closely with students' anticipated career paths, programs can enhance the relevance and applicability of research projects, reinforce professional identity, and provide clearer pathways to postgraduation employment.

Currently, the curriculum of the thesis courses evaluated in this study is structured to integrate independent research with guided mentorship, with a particular emphasis on the development of advanced skills in literature analysis and appraisal, experimental design and execution, and scientific communication (written and oral). Delivery is scaffolded through a sequence of assignments, regular checkpoints, and close interaction with faculty supervisors. Students complete a literature review relevant to their research project in the first term of the course, and laboratory supervisors formally assess student engagement, research execution, and responsiveness at three points during the year. The final deliverables for the course are a written thesis document and an oral thesis presentation. One striking finding was the increase in lab research as a post‐thesis career goal for biochemistry students (Fig. 5A). Therefore, future iterations of the course could incorporate workshops on advanced experimental methodologies, exposure to translational research settings, or navigating graduate school. This is a promising area of future exploration for our team.

### Limitations

A limitation of our study is the use of self‐reported survey tools, which are subject to social desirability bias as well as self‐selection bias [43, 44, 45]. To minimize the impact of social desirability bias, we made survey responses anonymous, along with making potentially identifiable qualitative questions optional. To minimize self‐selection bias, we used incentives to encourage participants among the target population. We also scheduled data collection times during lower‐stress periods during the academic year to encourage participation from a broad audience. Additionally, our present inquiry is limited to one cohort of students, and though the importance of richly described single‐cohort education studies has been discussed [46, 47], we must simultaneously be cautious when generalizing meaning to other contexts. Finally, it is worth noting that only 33 students completed both the pre‐ and postsurvey; there may be some perspectives or experiences that were not captured by our data.

## Conclusions

Overall, our findings contribute to the growing body of literature on the positive impacts of UREs, particularly within laboratory‐based senior thesis courses. To the best of our knowledge, this study is one of the few to holistically examine how UREs influence research skill development, epistemological beliefs, and career goals in a shared thesis setting across two distinct undergraduate programs. Notably, students reported significant gains in research self‐efficacy across multiple skill domains, including interpreting data, decoding scientific literature, visualizing data, and thinking like a scientist. These results underscore the powerful role of UREs in fostering core scientific competencies.

While there is remarkable potential for personal and professional growth through participating in a laboratory‐based senior research thesis, our results also revealed that development is not necessarily uniform and can be influenced by various contextual factors. This highlights opportunities for monitoring student growth, identifying barriers or gaps, and providing supplemental support. Taken together, these findings highlight the multifaceted benefits of UREs while emphasizing the importance of tailoring research experiences to meet the diverse developmental needs of students. Future research should focus on multicohort comparisons to disentangle cohort‐specific influences from more generalizable developmental trends. As UREs often mark students' first real entry point into scientific inquiry, continued evaluation of these experiences will be essential in designing inclusive, developmentally rich research opportunities that support both skill acquisition and identity formation in STEM.

## Conflict of interest

The authors declare no conflict of interest.

## Author contributions

CS conceived and designed the project. CS and HLZ acquired the data. CS, HLZ, and MO analyzed and interpreted the paper. CS and HLZ wrote the first draft, with MO, CEM, and FV providing revisions. CEM and FV provided supervisory support and project funding.

## Supporting information


**Table S1.** Pre‐thesis survey design, questions, and question response Formats. Questions analyzed with reverse score measures are indicated by (R).

**Table S2.** Post‐thesis survey design, questions, and question response formats. Questions analyzed with reverse score measures are indicated by (R).

## Data Availability

Deidentified quantitative data that support the findings of this study are available from the corresponding author (vulcuf@mcmaster.ca) upon reasonable request. Due to our small target population, information including qualitative data and combined demographic data makes participants potentially identifiable and is thus only available for total cohort description.
