# Supplementary material for: Impact of a senior research thesis on students' perceptions of scientific inquiry in distinct student populations
Source: FEBS Open Bio. 2025 Oct 25;16(3):595–609. doi: 10.1002/2211-5463.70145 (PMC12955745; doi:10.1002/2211-5463.70145)
Supplement: Supplementary file 1 — Table S1. Pre‐thesis survey design, questions, and question response Formats. Questions analyzed with reverse score measures are indicated by (R). [file FEB4-16-595-s001.docx]

**Supplementary Table 1: Pre-Thesis Survey Design, Questions, and Question Response Formats. Questions analyzed with reverse score measures are indicated by (R) [27,28].**

| **Question Section** | **Question** | **Question Response Format** | **Question Section** |
| --- | --- | --- | --- |
| **Survey ID Generation** | What are the first three letters of your eldest parent's first name? | Short free text | Yes |
|  | What are the first three letters of your youngest parent's first name? | Short free text | Yes |
|  | What are the first three letters of your birth month? | Short free text | Yes |
|  | What are the first three letters of the city you were born in? | Short free text | Yes |
| **Demographic Questions** | How would you describe your gender? | Short free text | Yes, with the option to not disclose by writing “N/A” |
|  | Please select which senior thesis course you are registered in. | List (BIOCHEM 4F09, BIOCHEM 4T15, BIOMEDDC 4A15) | Yes |
|  | What past laboratory research experience do you have? Please select ALL of the following statements that apply to you. | Multiple choice (I have not previously worked, volunteered, or had a project course in a research laboratory, I previously worked or volunteered in the lab I am currently doing my senior thesis in for eight weeks or longer, I did a third-year undergraduate thesis in a research laboratory (BIOCHEM 3A03, 3R06 or equivalent project course code), I have worked or volunteered in a research laboratory separate from my thesis placement for eight weeks or longer, Prefer not to disclose, Other) | Yes |
|  | Which of the following laboratory courses have you previously completed? Please select ALL of the following statements that apply to you. | Multiple choice (BIOCHEM 2L06: Inquiry in Biochemical Techniques, BIOCHEM 3LA3: Advanced Biochemistry Techniques, BIOMEDDC 3C09: Research Skills Laboratory and Inquiry, Another upper-year laboratory course (please describe in the text box below and indicate if the lab was completed online or in-person), Prefer not to disclose) | Yes |
|  | Please describe the other upper-year laboratory course you completed and indicate if the lab was completed online or in-person. | Long free text | Soft. Follow up question if student selected “Another upper-year laboratory course (please describe in the text box below and indicate if the lab was completed online or in-person)” in the previous question. |
|  | Which of the following categories do your career goals after your undergraduate degree fall into? If your career goal could fit into multiple categories, please select the description that fit the most closely to your career goals. | List (Unsure / I don’t know yet, Laboratory Research (Academia or Industry), Science or Health Science Fields (Medicine, Dentistry, Engineering, etc), Prefer not to disclose) | Yes |
| **Abilities, Attitudes, and Beliefs about Scientific Inquiry** | **Decoding Literature:** Please rate your agreement with the following statements. 1. **(R)** The scientific literature is difficult to understand. 2. I am confident that I could read a scientific paper and then explain it to another person. 3. I am confident in my ability to critically review scientific literature. 4. **(R)** When I see scientific journal articles it looks like a language I don’t understand. 5. **(R)** I am intimidated by the scientific language in journal articles. 6. I am comfortable defending my ideas about experiments. | Likert (Strongly Disagree, Disagree, I’m not sure, Agree, Strongly Agree) | Yes |
|  | **Interpreting Data:** Please rate your agreement with the following statements. 1. It is easy for me to transform data, like converting numbers from a table to percentages. 2. It is easy for me to relate the results of a single experiment to “the big picture.” 3. If I see data in a table, it is easy for me to understand what it means. 4. If I am shown data (graphs, tables, charts), I am confident that I can figure out what the data mean. | Likert (Strongly Disagree, Disagree, I’m not sure, Agree, Strongly Agree) | Yes |
|  | **Active Reading:** Please rate your agreement with the following statements. 1. I could make a simple diagram that provides an overview of an entire experiment.2. The way you display your data can affect whether or not people believe it. 3. If I am assigned to read a scientific paper, I typically look at the methods section to understand how the data were collected. 4. I know how to design a good experiment. | Likert (Strongly Disagree, Disagree, I’m not sure, Agree, Strongly Agree) | Yes |
|  | **Data Visualization:** Please rate your agreement with the following statements. 1. When I read scientific material it is easy for me to visualize the experiments that were done. 2. When I read scientific information, I usually look carefully at the associated figures and tables. 3. If I look at data presented in a paper, I can visualize the method that produced the data. 4. When I read a paper I have a clear sense of what physically went on in a lab to produce the results and information I am reading. | Likert (Strongly Disagree, Disagree, I’m not sure, Agree, Strongly Agree) | Yes |
|  | **Think like a Scientist:** Please rate your agreement with the following statements. 1. I enjoy thinking up additional experiments when I read scientific papers. 2. **(R)** I accept the information about science presented in newspaper articles without challenging it. 3. After I read a scientific paper, I think I could explain it to somebody else. | Likert (Strongly Disagree, Disagree, I’m not sure, Agree, Strongly Agree) | Yes |
|  | **Research in Context:** Please rate your agreement with the following statements. 1. Progress in curing many diseases has been made as a result of experiments on lower organisms like worms and flies. 2. I understand why experiments have controls. 3. Experiments in “model organisms” like the fruit fly have led to important advances in understanding human biology. | Likert (Strongly Disagree, Disagree, I’m not sure, Agree, Strongly Agree) | Yes |
|  | **Certainty of Knowledge:** Please rate your agreement with the following statements. 1. **(R)** Results that do not fit into the established theory are probably wrong. 2. **(R)** Because scientific papers have been critically reviewed before being published, it is unlikely that there will be flaws in scientific papers. 3. **(R)** The data from a scientific experiment can only be interpreted in one way. 4. **(R)** If two different groups of scientists study the same questions, they will come to similar conclusions. 5. Sometimes published papers must be reinterpreted when new data emerge years later. 6. **(R)** Because all scientific papers are reviewed by other scientists before they are published, the information in the papers must be true. | Likert (Strongly Disagree, Disagree, I’m not sure, Agree, Strongly Agree) | Yes |
|  | **Ability is Innate:** Please rate your agreement with the following statements. 1. **(R)** You must have a special talent in order to do scientific research. 2. **(R)** I think professionals carrying out scientific research were probably straight “A” students as undergrads. | Likert (Strongly Disagree, Disagree, I’m not sure, Agree, Strongly Agree) | Yes |
|  | Please rate your agreement with the following statements: 1. Science is a creative activity. 2. I have a good sense of what research scientists are like as people. 3. I have a good sense of what motivates people to go into research 4. **(R)** Scientists usually know what the outcome of their experiments will be. 5. Collaboration is an important aspect of scientific experimentation | Likert (Strongly Disagree, Disagree, I’m not sure, Agree, Strongly Agree) | Yes |
| **Open-ended Questions** | What are three things you hope to gain from your senior thesis this year? | Long free text | Optional |
